# Analysis of a zebrafish *dync1h1 *mutant reveals multiple functions for cytoplasmic dynein 1 during retinal photoreceptor development

**DOI:** 10.1186/1749-8104-5-12

**Published:** 2010-04-22

**Authors:** Christine Insinna, Lisa M Baye, Adam Amsterdam, Joseph C Besharse, Brian A Link

**Affiliations:** 1 Department of Cell Biology, Neurobiology and Anatomy, Medical College of Wisconsin, 8701 Watertown Plank Road, Milwaukee, WI 53226-0509, USA; 2 David H Koch Institute for Integrative Cancer Research, Massachusetts Institute for Technology, 77 Massachusetts Avenue E17-336, Cambridge, Massachusetts 02138, USA

## Abstract

**Background:**

Photoreceptors of the retina are highly compartmentalized cells that function as the primary sensory neurons for receiving and initiating transmission of visual information. Proper morphogenesis of photoreceptor neurons is essential for their normal function and survival. We have characterized a zebrafish mutation, *cannonball*, that completely disrupts photoreceptor morphogenesis.

**Results:**

Analysis revealed a non-sense mutation in *cytoplasmic dynein heavy chain 1 *(*dync1h1*), a critical subunit in Dynein1, to underlie the *cannonball *phenotypes. Dynein1 is a large minus-end directed, microtubule motor protein complex that has been implicated in multiple, essential cellular processes. In photoreceptors, Dynein1 is thought to mediate post-Golgi vesicle trafficking, while Dynein2 is thought to be responsible for outer segment maintenance. Surprisingly, *cannonball *embryos survive until larval stages, owing to wild-type maternal protein stores. Retinal photoreceptor neurons, however, are significantly affected by loss of Dync1h1, as transmission electron microscopy and marker analyses demonstrated defects in organelle positioning and outer segment morphogenesis and suggested defects in post-Golgi vesicle trafficking. Furthermore, dosage-dependent antisense oligonucleotide knock-down of *dync1h1 *revealed outer segment abnormalities in the absence of overt inner segment polarity and trafficking defects. Consistent with a specific function of Dync1h1 within the outer segment, immunolocalization showed that this protein and other subunits of Dynein1 and Dynactin localized to the ciliary axoneme of the outer segment, in addition to their predicted inner segment localization. However, knock-down of Dynactin subunits suggested that this protein complex, which is known to augment many Dynein1 activities, is only essential for inner segment processes as outer segment morphogenesis was normal.

**Conclusions:**

Our results indicate that Dynein1 is required for multiple cellular processes in photoreceptor neurons, including organelle positioning, proper outer segment morphogenesis, and potentially post-Golgi vesicle trafficking. Titrated knock-down of *dync1h1 *indicated that outer segment morphogenesis was affected in photoreceptors that showed normal inner segments. These observations, combined with protein localization studies, suggest that Dynein1 may have direct and essential functions in photoreceptor outer segments, in addition to inner segment functions.

## Background

Photoreceptors are highly polarized sensory neurons that require intense protein trafficking through a narrow connecting cilium to optimize phototransduction in the light sensitive outer segment [1,2]. As part of its normal physiology, the outer segment turns over about 10% of its length every day through a process called disc shedding. Disc shedding is compensated for by new outer segment assembly to maintain its length. The synthetic machinery supporting outer segment turnover is in the inner segment. In this compartment, newly synthesized outer segment proteins such as rhodopsin and phospholipids are delivered to the base of the connecting cilium in a vesicular fraction derived from the trans-Golgi [3,4]. The inner segment also supports the highly dynamic synaptic compartment via a short axon.

Microtubule based motors are thought to play a significant role in each of the major compartments of the photoreceptor. For example, axonal transport by both dynein and kinesin motors is necessary to support proper ribbon synapse formation and maintenance [5,6], and recent observations in zebrafish indicate that Dynactin1 is required for nuclear positioning in zebrafish [7]. Furthermore, Golgi and endoplasmic reticulum positioning and post-Golgi trafficking generally involve microtubule based motors [8,9], and outer segment turnover is now known to depend on intraflagellar transport using kinesin and dynein motors along the axoneme of the outer segment [1]. Thus, photoreceptors utilize multiple microtubule based motors in diverse cellular processes to facilitate normal development, maintenance, and function.

Dynein1 is a multi-subunit complex that consists of two 530 kDa heavy chains, responsible for force production, a group of 74 kDa intermediate chains, 53 to 57 kDa light intermediate chains, and 8 to 21 kDa light chains [10]. In photoreceptors, Dynein1 has been implicated in post-Golgi trafficking of rhodopsin because Dynlt1 (formerly Tctex-1), a dynein1 light chain subunit, binds to the carboxy-terminal domain of rhodopsin and can translocate rhodopsin-containing vesicles on microtubules [11]. However, Dynein1 has also been implicated in multiple cellular functions, including the positioning of the Golgi apparatus, endosomes, lysosomes, nuclei, centrosomes and mitotic spindles as well as retrograde axonal transport in neurons [9,12-15].

Another multi-subunit protein complex, Dynactin, serves as an adaptor and confers additional functions to Dynein1 by expanding the range of its cargo and increasing its motor processivity [13,14,16]. In a recent report, zebrafish embryos carrying a mutation (*mikre oko *or *mok*) in *dynactin1a *(*dctn1a*; previously called p150) failed to position photoreceptor nuclei to the proper layer without affecting the overall cell morphogenesis or the transport of opsins to the outer segment [7]. In the same study, the over-expression of another Dynactin component, Dctn2 (formerly p50/dynamitin), a manipulation known to dissociate the Dynein1/Dynactin complex, phenocopied *mok*. These results suggest that nuclear positioning in photoreceptor cells is carried out by a Dynein1/Dynactin dependent pathway.

To date, the precise cellular function(s) of Dynein1 in vertebrate photoreceptors remains poorly understood and recent studies have provided limited insight towards defining its role in trafficking and organelle positioning within the inner segment. In addition, potential Dynein1 functions within the outer segment have not been investigated. However, a second cytoplasmic Dynein, Dynein2, has been localized prominently in bovine photoreceptor outer segments along the connecting cilium, with expression also in the inner segment [17]. Functional insight for Dynein2 comes from knock-down studies in zebrafish where morpholinos directed against Dynein2 subunits resulted in short and disorganized photoreceptor outer segments [18]. Interestingly, the loss of Dynein2 function did not affect opsin trafficking, suggesting that Dynein2 plays a role in a transport pathway different from that proposed for Dynein1. Previous studies in *Chlamydomonas *and *Caenorhabditis elegans*, have clearly established a role for Dynein2 in the retrograde translocation of proteins from distal tips of ciliary axonemes to the base near the cell body [19-26]. This process, referred to as intraflagellar transport (IFT), requires a complex of at least 17 proteins for movement in the 'retrograde' direction. Indeed, in the study from Krock *et al*. [18], one anterograde IFT particle, IFT88, accumulated distally within photoreceptor outer segments of Dynein2 morphants, suggesting a failure in retrograde IFT.

Here we report the identification of a new zebrafish mutant, *cannonball *(*cnb*), which affects the *dynein cytoplasmic 1 heavy chain 1 *(*dync1h1) *locus. Using *cnb*, we studied the roles of Dynein1 in developing photoreceptor neurons. This mutant showed severe defects in photoreceptor organelle organization and outer segment formation. With regard to outer segment function, we present evidence that Dynein1 localizes along the connecting cilium and is detected in detergent extracted fractions enriched with axonemes of isolated outer segments. Finally, we used different concentrations of anti-sense morpholinos against *dync1h1 *to study dosage-dependent loss-of-function phenotypes. Embryos injected with a high dose of *dync1h1 *morpholino phenocopied *cnb *defects. Interestingly, lower concentrations of morpholino revealed specific outer segment defects. Overall, we provide support for multiple roles of Dynein1 in photoreceptor development, including organelle positioning, post-Golgi vesicle trafficking, and essential function(s) within the outer segment.

## Results

### The *cannonball *mutation affects photoreceptor development

**Figure 1:**
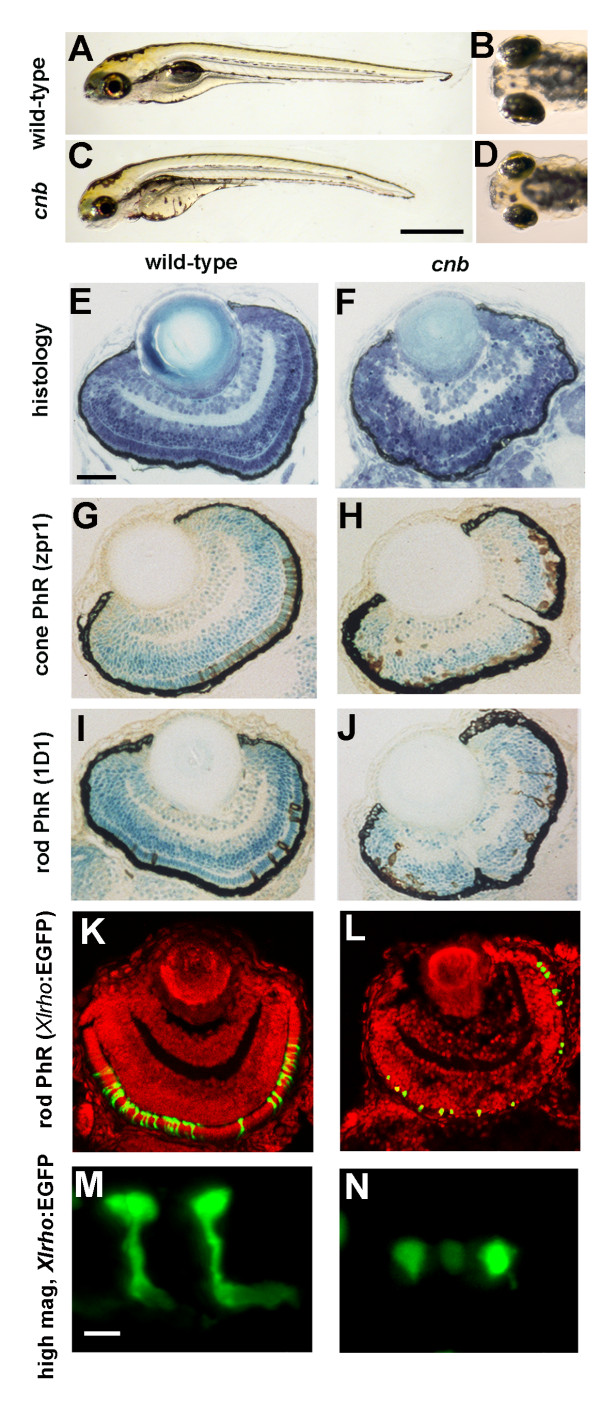
**The *cnb *mutation disrupts photoreceptor development**. **(A-D) **Side (A, C) and dorsal (B, D) views of 5-dpf wild-type (A, B) and *cnb *mutant (C, D) embryos. (**E-F**) Retinal histology of wild-type (E) and *cnb *(F) retina at 4 dpf. **(G, H) **Zpr-1 immunoreactivity (brown) to label double cone photoreceptors (PhR) in 4-dpf wild-type (G) and *cnb *embryos (H). **(I, J) **ID1 immunoreactivity (brown) to label rod photoreceptors in 4-dpf wild-type (G) and *cnb *embryos (H). Nuclei in (G, H) are counterstained with methylene blue. **(K-N) **Confocal microscopy of wild-type (K, M) and *cnb *(L, N) whole retina (K, L) and individual rod photoreceptor cells (M, N) from Tg(*Xlrho*:EGFP)^*fl*1 ^fish. Green, Xlrho:EGFP-positive cells; red, propidium iodide positive nuclei. Scale bars: 1 mm in (A, C); 40 μm in (E-L); 5 μm in (M, N). EGFP, enhanced green fluorescent protein.

The recessive larval lethal mutant *cnb *was isolated from a chemical mutagenesis screen for ocular defects in zebrafish. Embryos with the *cnb *mutation were indistinguishable from wild-type siblings until approximately 3.5 days post-fertilization (dpf), at which time the eyes were measurably smaller. By 5 dpf, the eyes were more obviously reduced in size. Mutant embryos died between 6 and 8 dpf, following rapid pan-cellular degeneration. In addition to eye defects, melanocytes often displayed an expanded morphology, giving embryos a darkened appearance (Figure 1A-D). Histology of the eyes revealed defects in photoreceptor cells, including a discontinuous outer plexiform layer and absence of obvious outer segments. The only photoreceptor cells that showed elongation were found in the central retina, where early born cells reside (Figure 1E-F). Immunohistochemical markers against cone (zpr1) or rod cells (1D1) indicated that both photoreceptor cell classes were generated, but lacked morphogenesis (Figure 1G-J). To investigate the photoreceptor defects more closely, we bred carriers of the *Tg(Xlrho:*EGFP)^*fl*1 ^transgene onto the *cnb *genotype. This transgene expresses cytoplasmic enhanced green fluorescent protein (EGFP) from the *Xenopus laevis *rhodopsin promoter and therefore highlights newly generated rod photoreceptors [27]. As has been previously described, confocal projections through wild-type photoreceptor cells in a *Tg(Xlrho:*EGFP)^*fl*1 ^retina highlights the cell body, inner segment and outer segment compartments [28] (Figure 1M). In comparison, *Xlrho*:EGFP-positive cells within *cnb *mutant retina were more spherical in shape and lacked segmental and elongated morphology (Figure 1N).

### Mutations in *dync1h1 *cause *cannonball *phenotypes

**Figure 2:**
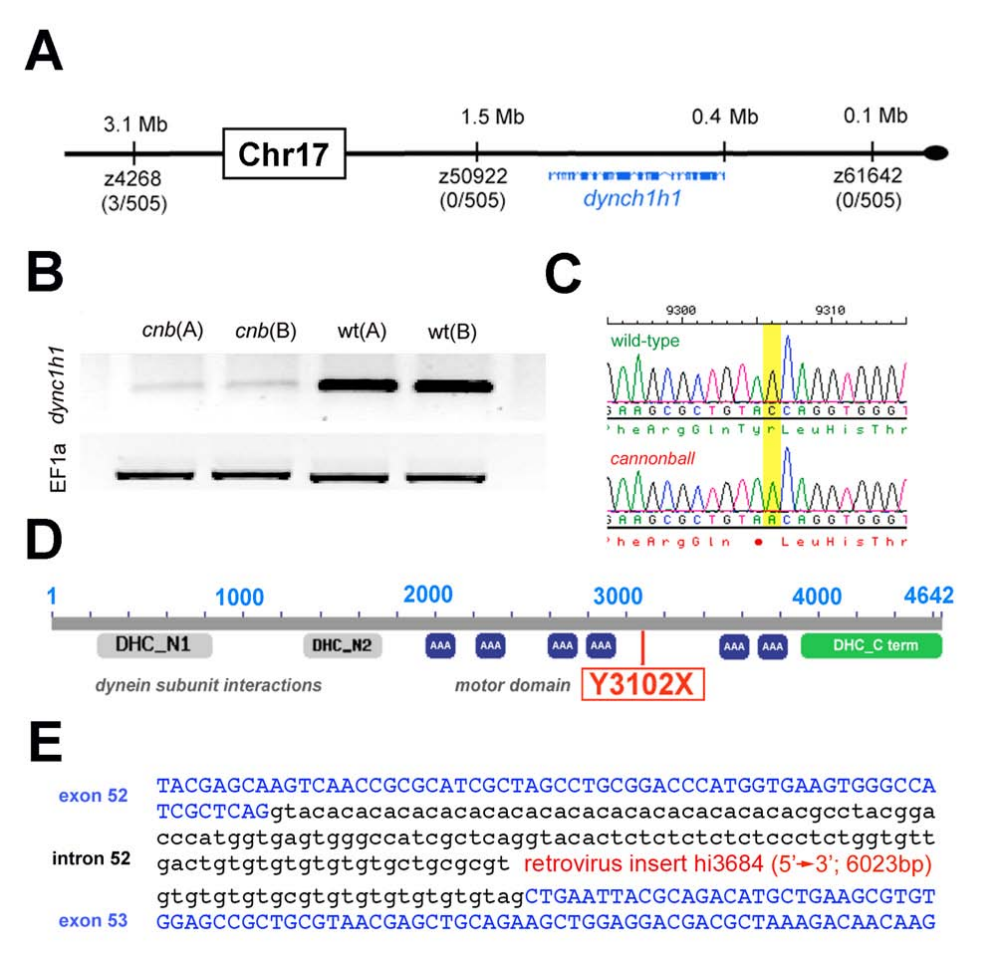
**The *cnb *locus encodes *dync1h1***. **(A) **Map of the *cnb *critical genomic region and exon/intron representation of *dync1h1*. Linked microsatellite markers and recombinants are placed onto the ZV7 zebrafish genomic map. **(B) **RT-PCR results from two independent cDNA samples each of *cnb *or wild-type embryos. Top image of bands from reactions with *dync1h1 *primers and bottom image of bands from reactions with *EF1alpha *primers. **(C) **Chromatograms showing wild-type (top) and *cnb *sequence flanking the C-to-A mutation. **(D) **Schematic of the Dync1h1 protein and location of the nonsense mutation. **(E) **Location of retrovirus insertion for *dync1h1 *mutant allele hi3684.

Recombinant linkage mapping positioned the *cnb *mutation near the end of chromosome 17 (Figure 2A). Analysis of public databases indicated the *dync1h1 *locus, along with other candidate genes, resided within the critical interval. To narrow the list of candidates, semi-quantitative RT-PCR was performed for each gene using cDNA from two pools of *cnb *embryos and two pools of wild-type sibling embryos. Of the transcripts analyzed within the mutant interval, only *dync1h1 *levels were reduced in *cnb *embryos (Figure 2B). Sequence analysis of *dync1h1 *genomic DNA from *cnb *embryos subsequently revealed a nonsense C-to-A mutation at nucleotide position 9,306 (Figure 2C). This mutation results in a substitution of a termination codon for the tyrosine at amino acid position 3,102 (Y3102X). Comparison of protein sequence between zebrafish and human Dync1h1 showed 91% identity and presence of all known functional domains. If translated, the *cnb *transcript is predicted to produce a truncated product that lacks the carboxy-terminal third of Dync1h1, including the stalk domain, essential for microtubule binding, as well as the fifth and sixth ATPase domains, essential for motor activity and overall structural integrity [29].

To confirm that this mutation causes *cnb *phenotypes, we performed a genetic complementation test with an insertional mutant of the *dync1h1 *gene. The hi3684 mutation was isolated as part of a larger scale mutagenesis screen for essential genes [30] and contains an insertion in the 52nd intron of the *dync1h1 *gene, after amino acid 3,397 (Figure 2E). Fish homozygous for the hi3684 mutation have phenotypes comparable to those of *cnb *mutants. Trans-heterozygous fish for the *cnb *and hi3684 alleles were indistinguishable from homozygous *cnb *mutant embryos, providing further evidence that mutations in *dync1h1 *cause the mutant phenotypes.

**Figure 3:**
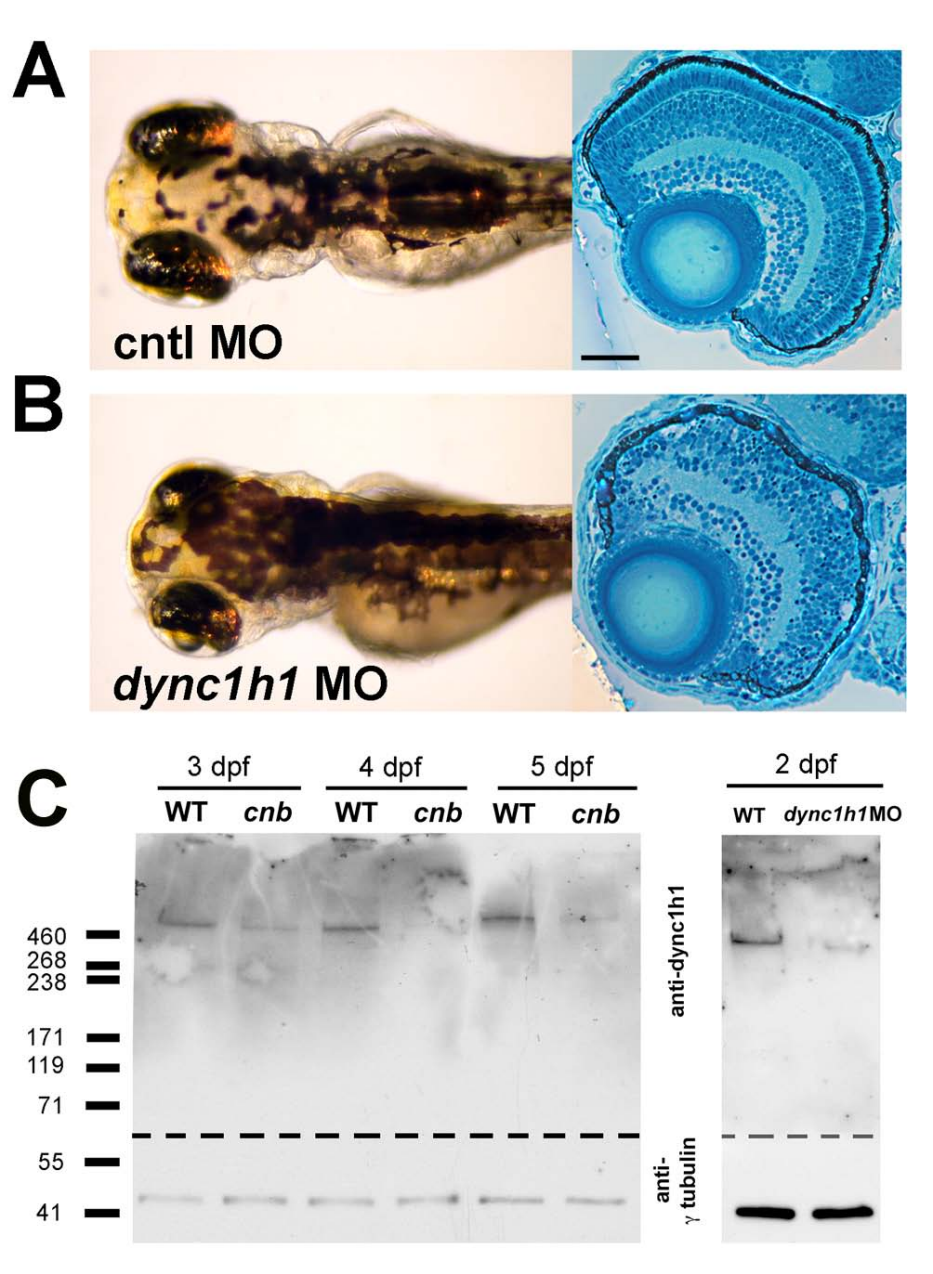
**Morpholino knock-down of *dync1h1 *phenocopies *cnb *mutants**. **(A, B) **Dorsal views and retinal histology of control (cntl) morpholino (MO) injected (A) and *dync1h1 *ATG MO injected (B) embryos shown at 3.5 dpf. Note the smaller eyes, expanded melanophores, and photoreceptor dysmorphogenesis. **(C) **Western blot of wild-type (WT) versus *cnb *embryos at 3, 4, and 5 dpf (left blot) and wild-type and *dync1h1 *morphants at 2 dpf (right blot). Each blot was cut and probed with either antisera against human DYNC1H1 (amino-terminal 321 amino acids; top) or gammaTubulin (bottom). Scale bar: 40 μm for retinal histology.

We next utilized an anti-sense oligonucleotide (morpholino) targeted against the start site of *dync1h1 *to further probe the *cnb *phenotype. Morpholino knockdown of *dync1h1 *resulted in a dose-dependent spectrum of phenotypes that overlap with those from *cnb *mutants (Figure 3A, B). Phenotypes were more severe in embryos injected with the highest dose of morpholino (8 ng/embryo). In these high-dose morphants, body axis curvature developed and the eyes were visibly smaller by 50 hpf, approximately 1 day before this phenotype was apparent in *cnb *mutants. These more severe phenotypes derived by knock-down using an ATG-morpholino suggest that wild-type maternal-derived RNA may compensate for the mutant transcript of *dync1h1 *during early embryogenesis in *cnb *fish. Indeed, RT-PCR indicated the presence of *dync1h1 *transcripts prior to the onset of zygotic transcription (data not shown).

Given the surprisingly mild embryonic phenotypes overall for predicted loss of *dync1h1 *function manipulations, we surmised that wild-type maternal-derived protein, in addition to mRNA, was also deposited in eggs, permitting normal development in many tissues during embryogenesis. To investigate this possibility, we performed western blots on *cnb *mutants and *dync1h1 *morphants from different ages (Figure 3C). Western blots and immunolocalization were carried out using an antibody directed against the amino-terminal 321 amino acids of human DYNC1H1 [17]. Indeed, at times when phenotypes were just beginning to show within the eyes of *cnb *mutant and *dync1h1 *morphant embryos, robust amounts of Dync1h1 protein were apparent. We did not detect truncated Dync1h1 protein in *cnb *mutants, indicating that the mutant transcript and/or protein may be unstable. Furthermore, the presence of wild-type-sized Dync1h1 protein in mutants is consistent with maternal-protein rescue of embryogenesis. In zebrafish, which maintain a large yolk plasm to cell ratio during development, this phenomenon of maternal rescue often results in mutants of cell-essential genes with surprisingly mild phenotypes [31,32].

**Figure 4:**
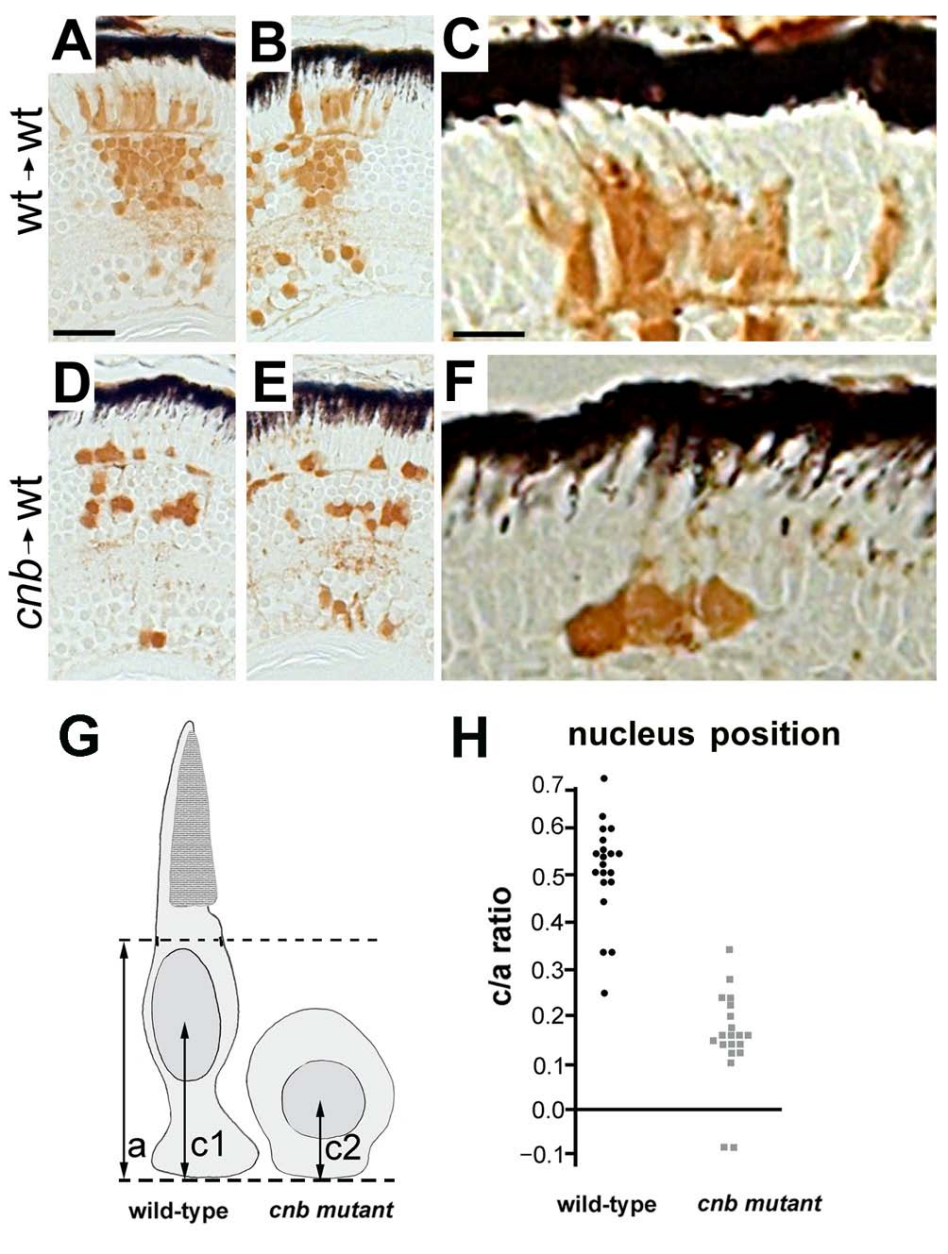
**Photoreceptors from *cnb*/*dync1h1 *mutants show cell-autonomous defects in morphogenes**. **(A-F) **Images of histology sections from genetically mosaic retinas of wild-type (wt) donor, wild-type host (A-C) or *cnb *donor, wild-type host (D-F). Two examples are shown at low magnification to indicate overall clone size and one example at higher magnification to show photoreceptor morphology. Donor cells are label with the brown lineage tracer. Black retinal pigment epithelium cells provide reference for orientation. Note the lack of elongated morphology in *cnb *photoreceptor cells. Scale bars: 40 μm in (A, B, D, E); 15 μm in (C, F). **(G) **Schematic showing how nuclear position was evaluated. Dashed lines represent the external limiting membrane (upper) and apical side of the outer plexiform layer (bottom) for the host retina. **(H) **Scatter plot of nuclear position (c/a ratio) for donor cells of wild-type (black, left side) or *cnb *(grey, right side) genotypes.

Previous studies in zebrafish have shown that mutations in Dynactin subunits, which complex to activate many of Dynein's functions, also show photoreceptor defects [7,33,34]. However, the photoreceptor dysmorphogenesis in *dctn1a *mutants appear less severe than that observed in *cnb *mutants. In addition, for the *mok*/*dctn1a *mutation, outer segment morphogenesis was completely restored when mutant cells are juxtaposed to healthy wild-type cells within genetic mosaic embryos. In those *mok *mutant cells, however, nuclear position was displaced toward the synaptic region and the photoreceptor cells eventually died [7]. To evaluate whether *cnb *photoreceptor cell morphology was rescued within a wild-type environment, we generated genetic mosaics by blastulae transplantation. While wild-type donor cells elaborated extensive columnar clones that produced normal-appearing photoreceptor cells, *cnb *donor cells produced smaller cell columns with abnormal photoreceptor neurons (Figure 4). All *cnb *photoreceptors, despite residing within a wild-type retina, lacked elongated morphology and retained their mutant appearance (compare Figure 4F to Figure 1N). In addition, we scored the position of the nucleus relative to the adjacent host cells, in a similar manner to how *mok *mutant nuclei were scored (Figure 4G) [7]. Like *mok *mutants, *cnb *nuclei were positioned closer to the synaptic region in wild-type host retina (Figure 4H).

**Figure 5:**
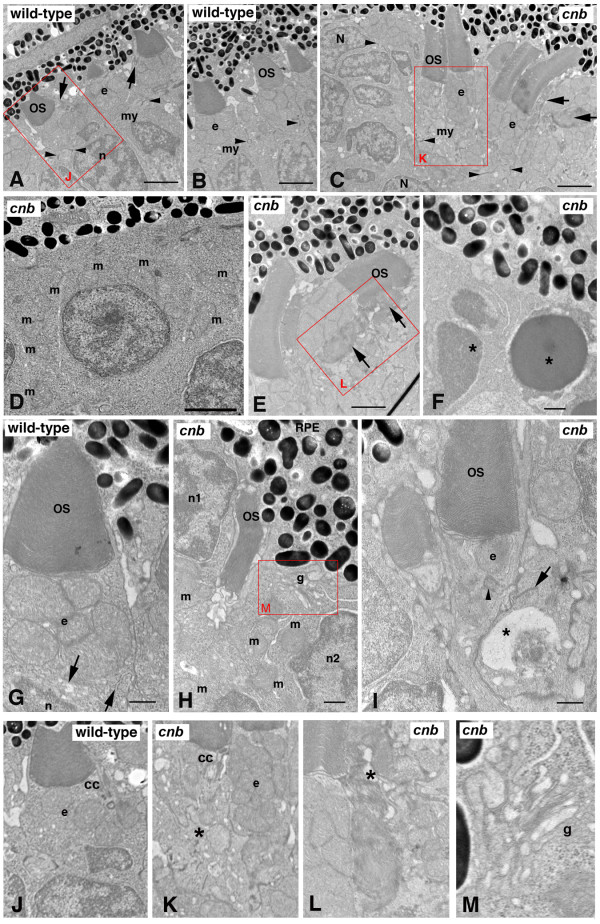
**The *cnb *mutation disrupts photoreceptor polarity and outer segment formation**. **(A, B) **TEM of a wild-type central retina at 3 dpf showing normal structure of outer segments (OS) with aligned discs adjacent to the ellipsoid (e) region of the inner segments with bundles of tightly compacted mitochondria. The myoid (my) of the inner segment is the region between the mitochondria-rich ellipsoid and the elongated nuclei (n). Dense adherens junctions (arrowheads) can be seen at the external limiting membrane. Arrows in (A) point to connecting cilia. See also (J) for high magnification. **(C-F, H, I) **TEM of *cnb *mutant retina. (C) TEM of a *cnb *central retina at 3 dpf with both normal photoreceptor outer segments (left) and cells with disorganized outer segments (right, arrows). Adherens junctions are indicated by arrowheads. Overall, photoreceptors in the peripheral retina showed more severe defects, including cells that failed to form a polarized inner segment and cells with pyknotic nuclei. (D) Higher magnification of peripheral *cnb *photoreceptors that lack polarity and display dispersed mitochondria (m). (E) Normal *cnb *photoreceptors (left) next to an abnormal cell with a disrupted outer segment (arrows). See (L) for higher magnification. (F) Photoreceptors at the periphery with pyknotic nuclei (asterisks) and dense granular cytoplasm. **(G) **High magnification image of polarized wild-type cone photoreceptor at 3 dpf showing normal outer segment, typical ellipsoid with clustered mitochondria, and a narrow myoid with membrane vesicles and cisternae (arrows). (H, I) Central cone photoreceptors of *cnb *mutant eyes showing abnormal polarity. In (H), the cell on the left labeled n1 has its ellipsoidal mitochondria (m) on the opposite side of the nucleus from the retinal pigment epithelium (RPE) with an adjacent miss-aligned outer segment. The cell on the right (n2) has its Golgi complex (g) adjacent to RPE and the mitochondria (m) is scattered throughout the cytoplasm. A higher magnification of the Golgi is shown in (L). In (I), a *cnb *cone photoreceptor with a normal OS has a single mitochondrion at the position of the ellipsoid (e) and a displaced centriole (arrowhead). The adjacent cell on the right displays an abnormally positioned synaptic region with a large vacuole (asterisk) and a synaptic ribbon (arrow) adjacent to the inner segment of the cone cell in the middle. **(J-M) **Higher magnification insets of the red boxed regions highlighting the connecting cilium (cc) and ellipsoid (e) regions. Asterisk in (K) indicates a cell with a disorganized inner segment adjacent to a cell with a more normal ellipsoid. Asterisk in (L) indicates disorganized outer segment. Scale bars: 2 μm in (A-C); 0.5 μm in (F-I).

To better evaluate the effects of the *cnb *mutation on photoreceptor development, we performed transmission electron microscopy (TEM) analysis on 3-dpf *cnb *embryos and their wild-type siblings (Figure 5; Tables 1 and 2). At this developmental time in the zebrafish retina, most photoreceptor cells are cones. By 3 dpf, wild-type cone photoreceptors typically have extended short outer segments that measure between 2 and 5 μm in length [35]. In *cnb *mutant retina, photoreceptor cells were positioned in the correct outer retinal layer; however, their nuclei lacked elongated morphology and only a few central retinal cells showed outer segments (Figure 5C, E, I). In fact, many cells also lacked inner segments. The absence of inner segment formation was accentuated in later born photoreceptors immediately peripheral to the most central retina. Furthermore, the number of pyknotic nuclei within the outer nuclear layer increased towards the retinal periphery (Figure 5F, asterisks). The gradient in severity of phenotypes from central to peripheral is consistent with the notion that wild-type maternal protein becomes more depleted in peripheral progenitor cells that had undergone more cell divisions prior to producing photoreceptor neurons.

**Table 1:** Specimens analyzed for ultrastructure

Sample	Eyes (*n*)	Fields of view (*n*)	Cells (*n*)
**Central**			
Control MO	4	11	132
*dync1h1 *MO	3	9	101
*cnb *mutant	4	12	139
			
**Peripheral**			
Control MO	4	9	111
*dync1h1 *MO	4	10	103
*cnb *mutant	4	9	108

MO, morpholino.

**Table 2:** Ultrastructure morphometrics

	Cell area (average ± SEM)		OS morphology		Cellular phenotypes			
			
Specimen	PhR area (μm^2^/cell)	OS area (μm^2^/cell)	Normal (% OS)	Vesiculated (% OS)	Polarized with OS (%)	Polarized no OS (%)	Depolarized cell (%)	Apoptotic nucleus (%)
**Central**								
Cntrl MO	22.0 ± 10.5	4.4 ± 0.3	89	11	64	36	0	0
*dync1*MO	20.9 ± 9.6	3.3 ± 0.2*	70^†^	30^†^	30^†^	49^†^	17^†^	5^†^
*cnb*	19.8 ± 13.2	3.5 ± 0.3*	86	14	16^†^	37^†^	32^†^	15^†^
								
**Peripheral**								
Cntrl MO	21.7 ± 14.6	4.0 ± 0.3	88	12	47	53	0	0.01
*dync1*MO	19.9 ± 12.5	3.0 ± 0.5*	60^†^	40^†^	5^†^	47^†^	40^†^	9^†^
*cnb*	20.0 ± 11.2	2.6 ± 0.6*	75^†^	25^†^	4^†^	14^†^	62^†^	20^†^

*P < 0.05 versus control morpholino (Cntrl MO) in central or peripheral regions (Student's *t*-test).

^†^*P *< 0.05 versus cntrl MO in central or peripheral regions (*X*^2 ^test). OS, outer segment; PhR, photoreceptor; SEM, standard error of the mean.

Because outer segments were absent in most *cnb *photoreceptor neurons, we focused our analysis on the inner segment. Multiple cell polarization and organelle positioning defects were noted in *cnb *photoreceptor cells. First, numerous mitochondria were mislocalized away from the characteristic bundle defining the ellipsoid region. The bundle also appeared less compact and isolated mitochondria were seen in the vicinity of nuclei (Figure 5G-K). Second, centrioles were often misoriented and separated from the basal body region (Figure 5I). Occasionally, centrioles were surrounded by mitochondria within the ellipsoid. Interestingly, in some centrally located photoreceptor cells that had normal inner segments, outer segment morphology was abnormal (Figure 5E, H, L). Overall, ultrastructural analysis of *cnb *photoreceptors revealed a spectrum of phenotypes that increased in severity from central to peripheral retina. A small number of central *cnb *photoreceptor cells were completely normal. Greater numbers of central photoreceptors showed defects specifically in the outer segments. The most common photoreceptor phenotype was a general disruption of organelle polarization within the inner segment region. Mutant photoreceptor cells in the periphery showed more significant polarity and organelle positioning defects, never formed outer segments, and often had pyknotic nuclei.

TEM analysis of embryos at 4.5 dpf revealed more severe phenotypes, including the complete absence of inner and outer segments in all cells analyzed (data not shown). Overall, the severity of developmental defects and rapid degeneration of outer segments caused by the *cnb *mutation precluded an extensive analysis of the photoreceptor phenotypes at later developmental stages.

### Dynein1 localizes to photoreceptor outer segments

**Figure 6:**
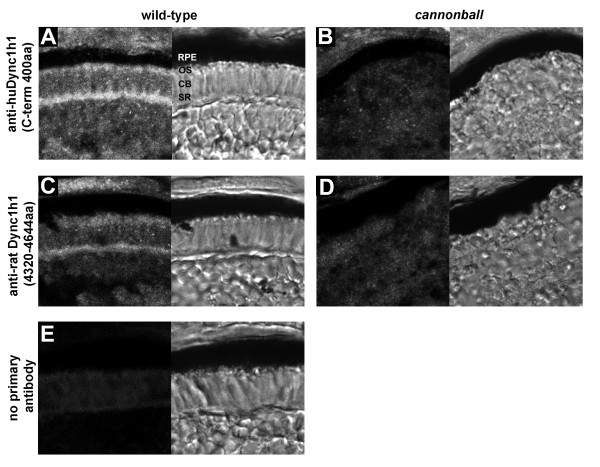
**Immunolocalization of Dync1h1 in developing photoreceptors**. **(A-E) **Cryosections of the photoreceptor layer from wild-type retina (A, C, E) or *cnb *mutant retina (B, D) each at 4 dpf. Antisera directed against two different regions of Dync1h1 was used: (A, B) anti-human DYNC1H1 (huDYNC1H1; carboxy-terminal 400 amino acids) and (C, D) anti-rat Dync1h1 (amino acids 4,320 to 4,644). Primary antibodies were omitted in (E). For each panel, the fluorescent image is shown on the left and the transmitted light image on the right. CB, cell body; OS, outer segment; RPE, retina pigment epithelium; SR, synaptic region.

The identification of *cnb *photoreceptor cells with relatively normal inner segments, yet abnormal outer segments, raised the question of whether Dynein1 functions within both the inner and the outer segment. We therefore characterized the subcellular distribution of Dynein1 subunits in photoreceptor cells. We first examined the localization of Dync1 h1 by immunostaining cryosections of 4-dpf wild-type or *cnb *mutant embryos (Figure 6). Dync1h1 was expressed widely, as expected, but showed enrichment in photoreceptors at the synaptic region and within and proximal to the developing outer segments. Three independent antisera showed equivalent staining and, importantly, immunoreactivity was significantly diminished in *cnb *mutant retina (Figure 6 and data not shown). The amino acid alignments for the immunogens (rat or human protein) and the zebrafish protein are shown in Additional file 1.

**Figure 7:**
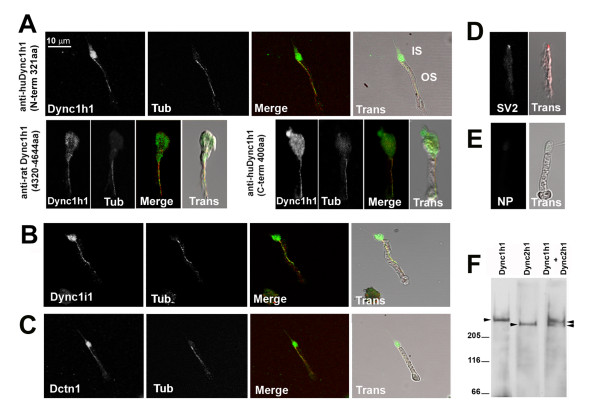
**Dync1h1, Dync1i1, and Dnct1 immunoreactivity is associated with photoreceptor axonemes**. Immunocytochemical localization of proteins in isolated adult zebrafish photoreceptor cells. **(A) **Immunocytochemical localization of Dynein1 heavy chain (Dync1h1) using antisera directed against three different epitopes. **(B) **Immunocytochemical localization of Dynein1 intermediate chain (Dync1i1), **(C) **Dynactin1/p150 (Dctn1), and **(D) **Synaptic vesicle protein 2 (SV2). **(E) **Immunostaining in which the primary antibody was omitted. In (A-C), cells were co-stained with acetylated α-tubulin (Tub) to highlight the axoneme. Color merged (Merge) and transmitted light images overlaid with Merge images (Trans) are shown for each panel. IS, inner segment; OS, outer segment. NP, no primary antibody. **(F) **Western blot analysis on detergent-extracted photoreceptor cytoskeleton (DEPC) fraction shows the specificity of the Dync1h1 antibody, as the Dync2 h2 antibody recognizes a discrete and faster migrating band.

To better visualize subcellular location, isolated photoreceptor preparations from adult zebrafish were probed with the antibodies to Dync1h1 or other Dynein1-associated proteins (Figure 7A-E). These isolated preparations have previously been used for detection of IFT motor proteins in the outer segment [36]. In agreement with prior work [11], all three Dync1h1 antibodies showed abundant staining in the inner segment (Figure 7A). In addition, strong, punctate immunoreactivity was also detected along the entire length of the ciliary axoneme within the outer segment. Double labeling performed with an anti-α-acetylated tubulin antibody, a marker for axonemal microtubules, confirmed that Dynein1 co-localizes with the axoneme. Furthermore, antibodies against Dynein1 intermediate chain (Dync1i1) and Dynactin1 (Dynct1) showed identical patterns of localization to Dync1h1 (Figure 7B, C). The lack of outer segment immunoreactivity against a synaptic vesicle protein, SV2, confirmed the specificity of the axomemal localization of Dynein components (Figure 7D).

Prior studies in mouse and zebrafish photoreceptors described a potential role for another cytoplasmic dynein, Dynein2, in retrograde IFT along the connecting cilium [17,18]. In order to further validate whether our Dync1h1 antibodies are specific and do not cross-react with Dync2 h1 of Dynein2, we used bovine retina to make a detergent-extracted photoreceptor cytoskeleton (DEPC) fraction. Immunoblotting with this fraction, which is enriched in connecting cilia, showed the presence of both Dynein1 and Dynein2 heavy chains (Figure 7F). When used separately in DEPC fractions, the antibodies against either Dync1h1 or Dync2 h1 generated single bands at the expected sizes (530 and 450 kDa, respectively). Furthermore, these two bands were distinguishable when extract was probed simultaneously with both antibodies. Cumulatively, these data show that, in addition to Dynein2, Dynein1 also associates with the ciliary axoneme and potentially has direct outer segment functions. Our observations are also consistent with recent proteomic analyses of purified mouse and bovine photoreceptor outer segments and cilia [37,38].

### Dose-sensitive effects of Dynein1 knock-down on outer segment formation

**Figure 8:**
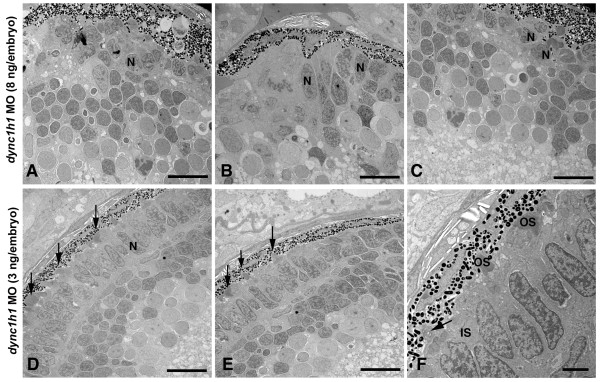
**Knock-down of Dync1h1 phenocopies *cnb *mutants**. **(A-C) **TEM of retinas from 3-dpf embryos injected with a high dose (8 ng/embryo) of *dync1h1 *morpholino (MO). Photoreceptors across the entire retina failed to form outer segments and had rounded nuclei (N). **(D-L) **TEM views of embryos injected with a lower amount (3 ng/embryo) of *dyn1 hc1 *morpholino. Outer segments formed in some photoreceptors at the central (D, E) and peripheral retina (F) (arrows). Most lower dose morphant cells still failed to elongate outer segments. Scale bars: 10 μm in (A-E); 2 μm in (F).

**Figure 9:**
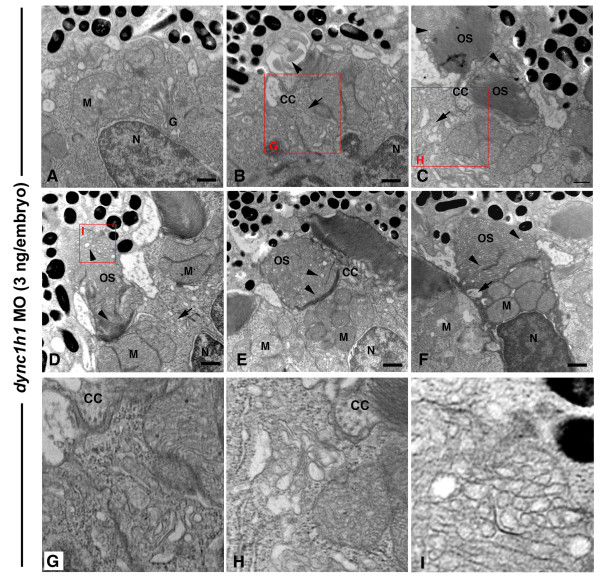
**Dync1h1 knock-down causes dose-sensitive outer segment defects**. TEM of retinas from 3-dpf embryos injected with a high dose (8 ng/embryo) of *dync1h1 *morpholino (MO). **(A) **A cone cell with Golgi apparatus (G) localized in the central ellipsoid region and mitochondria (M) located near the nucleus (N). **(B-I) **Cone cells with accumulation of vesicles (arrows) at the base of the connecting cilium (CC) and presence of disorganized disc structures within the outer segment (OS; arrowheads). **(D-F) **Severely disrupted outer segments showing vesiculation and tubulation of discs structures distally (arrowheads), yet normal discs at the base. Large vacuoles and vesicle accumulations were found at the base of the connecting cilium (arrows). The condensed cell in (F) is consistent with the morphology of early stage apoptosis. **(G-I) **Higher magnification insets of the red boxed regions. Scale bars: 0.5 μm in (A-F).

**Figure 10:**
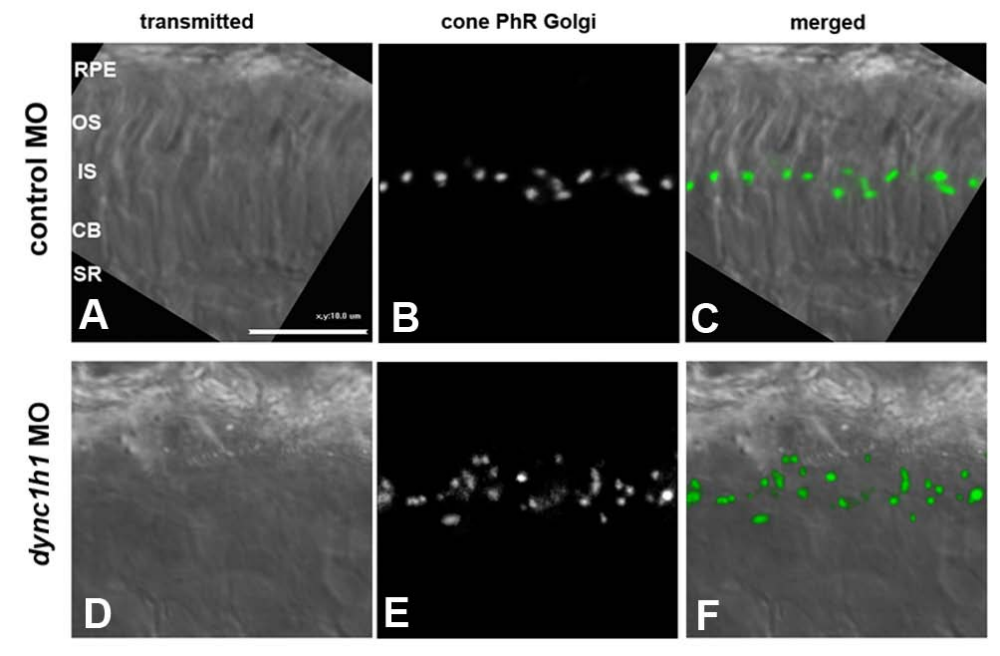
**Localization of Golgi apparatus in *dync1h1 *morphants**. **(A-F) **Expression of a tol2-based *gnat2:*Man2a(1-100aa)-GFP construct in either control morphants (3 ng/embryo) (A-C) or *dync1h1 *morphants (3 ng/embryo) (D-F). Cryosections of the photoreceptor (PhR) layer at 4 dpf is shown. Embryos were treated with phenylthiourea to block pigment synthesis. Note that disorganization and mis-positioning of the Golgi apparatus in *dync1h1 *morphants. CB, cell body; IS, inner segment; MO, morpholino; OS, outer segment; RPE, retina pigment epithelium; SR, synaptic region.

While TEM analysis of the *cnb *mutants provided valuable insight into the requirement of Dynein1 for organelle positioning, the paucity of mutant photoreceptor cells with outer segments made detailed characterization within that cellular compartment difficult. The central to peripheral gradient in phenotype severity suggested that different degrees of *dync1h1 *hypomorphism resulted in distinct subcellular phenotypes, including cells with outer segment defects. We therefore used different doses of *dync1h1 *morpholinos to investigate ultra-structural consequences to varied levels of gene knockdown. Embryos injected with a high dose (8 ng/embryo) of *dync1h1 *morpholino resulted in photoreceptor phenotypes very similar to the *cnb *mutants (Figure 8A-C). In low dose morphants (3 ng/embryo), photoreceptor cells showed less severe phenotypes, including a greater number with some outer segment formation (Figure 8D-F; Tables 1, 2, and 3). However, many of these morphant photoreceptor cells displayed organelle polarization defects within the inner segment. Although the Golgi apparatus appeared morphologically intact in every cell, it was occasionally disorganized and accompanied by abnormal amounts of vesicles at the ellipsoid region and at the base of the connecting cilium (Figure 9A-C, G, H). To further address Golgi organization in *dync1h1 *morphants, we expressed a transgene (-*3.2 kb gnat2: *Man2a(1-100aa)-GFP) that encodes a fusion protein that specifically localized to the Golgi of cone photoreceptor cells (Figure 10). In control morphants, fluorescence was found in a position just apical to the nucleus within the myoid of cone cells. In low dose *dync1h1 *morphants, fluorescence was less regular and often scattered throughout the cell body.

**Table 3:** Summary of photoreceptor phenotypes associated with loss of Dynein1 or Dynactin

Genotype	Rounded nuclei	Cells lacking inner segments	Organelle position defects	Outer segment defects
*mok*/*dctn1a*	Yes	No	Nuclei, mitochondria (mild)	Short OS
*dctn1a+1b *MO	Yes	No	Nuclei, mitochondria, Golgi (mild)	Short OS
*cnb*/*dync1h1*	Yes	Yes	Nuclei, mitochondria, Golgi, centrioles (severe)	Short, disorganized OS prone to vesiculation, total lack of OS
*dync1h1 *MO (high dose)	Yes	Yes	Nuclei, mitochondria, Golgi, centrioles (severe)	Short, disorganized OS prone to vesiculation, total lack of OS
*dync1h1 *MO (low dose)	Yes	No	Mitochondria, Golgi, centrioles (mild)	Short, disorganized OS prone to vesiculation

MO, morpholino; OS, outer segment.

Although outer segments were formed, they were often dramatically disorganized and vesiculated in low dose *dync1h1 *morphants. Even in morphant cells with apparently normal inner segments, the distal outer segments frequently filled up with vesicle-like structures and the discs lost their normal shape and orientation (Figure 9B-F, I).

**Figure 11:**
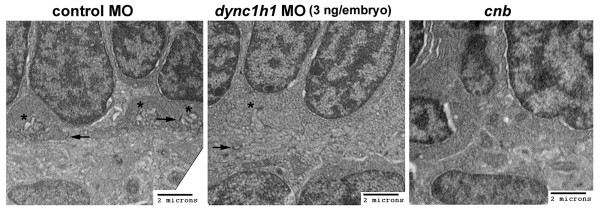
**Ultrastructure of synaptic region in *cnb *mutants and *dync1h1 *morphants**. TEMs of the photoreceptor synaptic region and outer plexiform layer of 3-dpf embryos. **(A) **Control morphants (3 ng/embryo) showed well developed synaptic input invaginations (asterisks) and numerous floating ribbons (arrows). **(B) ***dync1h1 *morphants (3 ng/embryo) had less defined synaptic input invaginations (asterisk) and significantly reduced number of floating ribbons (arrow). **(C) ***cnb *mutants did not show synaptic input invaginations and typically lacked ribbons. MO, morpholino.

Finally, *dync1h1 *morphants, as well as *cnb *mutant cells, did not form proper synaptic terminal structures and lacked the characteristic membrane invaginations, indicating that they were disconnected from post-synaptic regions of bipolar and horizontal cells (Figure 11).

### Knock-down of Dynein1 and Dynactin subunits suggest differential requirement for outer segment development

We next explored the role of Dynactin in contributing to the photoreceptor defects due to loss of Dynein1 function. The lack of outer segment defects early in *mok*/*dctn1a *mutant development implies that Dynein1 may function independently from Dynactin in the outer segment [7,33]. Consistent with this possibility, the binding of Dynactin1 to Dynein1 has been reported as non-essential for the ability of Dynein1 to bind stably to rhodopsin transport vesicles *in vitro *[11]. However, an alternative possibility is that loss of *mok*/*dctn1a *function is compensated by *dynactin1b *(*dctn1b*), a duplicated, highly similar paralog (77% identical to *dctn1a*) that is unique to teleost fish [7]. Consistent with a potential role for Dynactin1 in outer segment function, we found immunoreactivity for this protein along the axoneme (Figure 7C).

**Figure 12:**
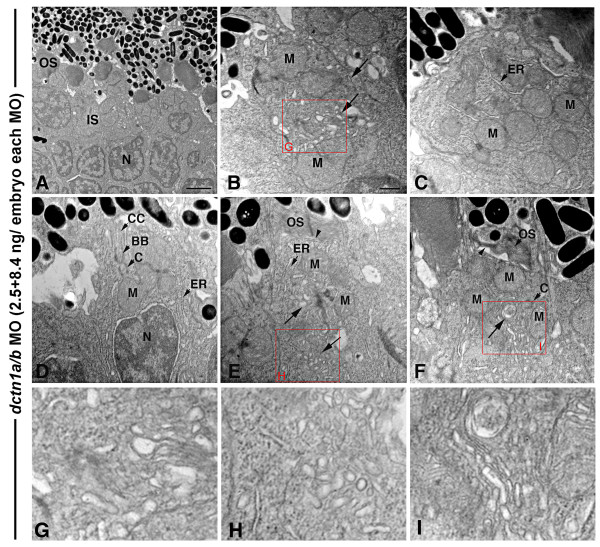
**Double *dnct1a*/*b *knock-down primarily affects the inner segment**. **(A) **TEM view of the central retina of a *dctn1a*/*b *morphant at 3 days showing normal, albeit short, outer segment structures. (**B-F**) TEM views of individual photoreceptors at the peripheral *dctn1a*/*b *morphant retina with inner segment polarization defects, including a dispersion of mitochondria (M) within that region (B, C, F), lack of a distinct myoid region (D) and positioning of the ER/Golgi complex among dispersed mitochondria (arrows in (B, E, F)). In addition, accumulation of Golgi associated vesicles in the inner segment (arrow in (B, E, F)) and shortened outer segment structures (arrowheads) were also seen (E, F). **(G-I) **Higher magnification insets of the red boxed regions. For morpholino (MO) injections, *dnct1a *splice MO was used at 2.5 ng/embryo, while the *dnc1b *splice MO was used at 8.4 ng/embryo (for a total MO concentration of 10.9 ng/embryo). BB, basal body; C, centriole; CC, connecting cilium; ER; endoplasmic reticulum; IS, inner segment; M, mitochondria; OS, outer segment. Scale bar: 2 μm in (A); 0.5 μm in (B-F).

To test the potential function of Dynactin1 in the outer segments, we used morpholinos to simultaneously knock-down both *dctn1a *and *dctn1b*. A *dctn1a *morpholino has been previously described [39] and we generated a splice site morpholino against *dctn1b *that resulted in destabilization of the transcript (Additional file 2). Use of a translation inhibiting *dctn1b *morpholino gave equivalent phenotypes (data not shown). TEM analysis of embryos co-depleted for *dctn1a *and *dctn1b *(*dctn1a*/*b *morphants) revealed that most photoreceptors had either completely normal or slightly shorter outer segments, neither of which showed disc morphology defects. However, abnormal positioning of the Golgi complex and mitochondria within inner segments was noted in *dctn1a*/*b *morphants (Figure 12, Table 3). As described for *cnb *mutants and *dync1h1 *morphants, but to a milder degree, these organelles were mislocalized and/or misoriented. In particular, morphant cells often failed to localize mitochondria within a discrete ellipsoid region (Figure 12A-F). In addition, significant accumulations of vesicles were found in association with Golgi complexes in the inner segments, suggesting deficiencies in post-Glogi vesicular transport (Figure 12G-H). Because vesicle accumulation was only rarely observed at the base of the connecting cilium, we were unable to conclude whether or not the transport of post-Golgi vesicles towards the outer segment was affected.

Overall, simultaneous knock-down of both *dctn1a *and *dnct1b *genes produced phenotypes stronger than *mok*/*dctn1a *alone, but much less severe than loss of Dynein1 function. In *dctn1a*/*b *morphants, inner segment organelle and polarity defects were noted, but the only outer segment phenotype observed was reduced length in some photoreceptor cells, which could be attributed to inner segment defects.

**Figure 13:**
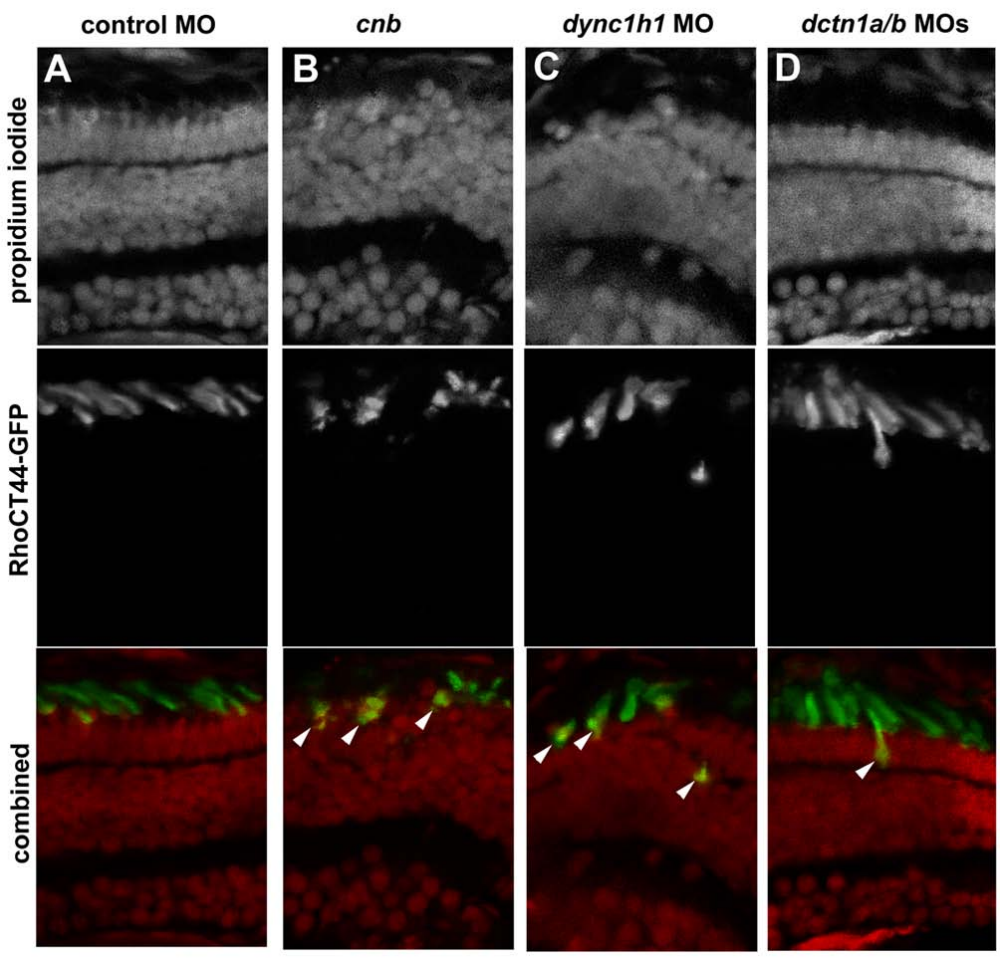
**Efficient transport of Rhodopsin(CT44)-GFP to the outer segment is disrupted with loss of Dync1h1 or Dctn1**. Cryosections of 3.5-dpf retina from transgenic Tg(1.3*xops*:xRhoCT44-GFP) embryos in which the carboxy-terminal 44 amino acids of Rhodopsin are fused with GFP. Localization of GFP was evaluated in photoreceptors from **(A) **control morphants, **(B) ***cnb *mutants, **(C) ***dync1h1 *morphants or **(D) ***dctn1a*/*b *morphants. Upper row shows propidium iodide staining to highlight nuclei. Middle row shows RhoCT44-GFP. Bottom row shows color combined images. Arrowheads indicate cells with mislocalized GFP to the cell body region.

In a final series of experiments, we explored whether opsin-based trafficking was affected with loss of either Dynein1 or Dynactin. To investigate this, we used the Tg(1.3*xops*:xRhoCT44-GFP) transgenic line [40]. Fish with this transgene express GFP fused with the carboxy-terminal 44 amino acids of Rhodopsin; this fusion protein is transported to the outer segment with high efficiency (Figure 13A). In both *cnb *mutant and *dync1h1 *morphant cells, however, we found multiple examples of mislocalized RhoCT44-GFP (Figure 13B, C). In addition, *dctn1a*/*b *morphant retina also showed RhoCT44-GFP transport defects, but at a proportion lower than loss of *dync1h1 *(Figure 13D).

## Discussion

**Figure 14:**
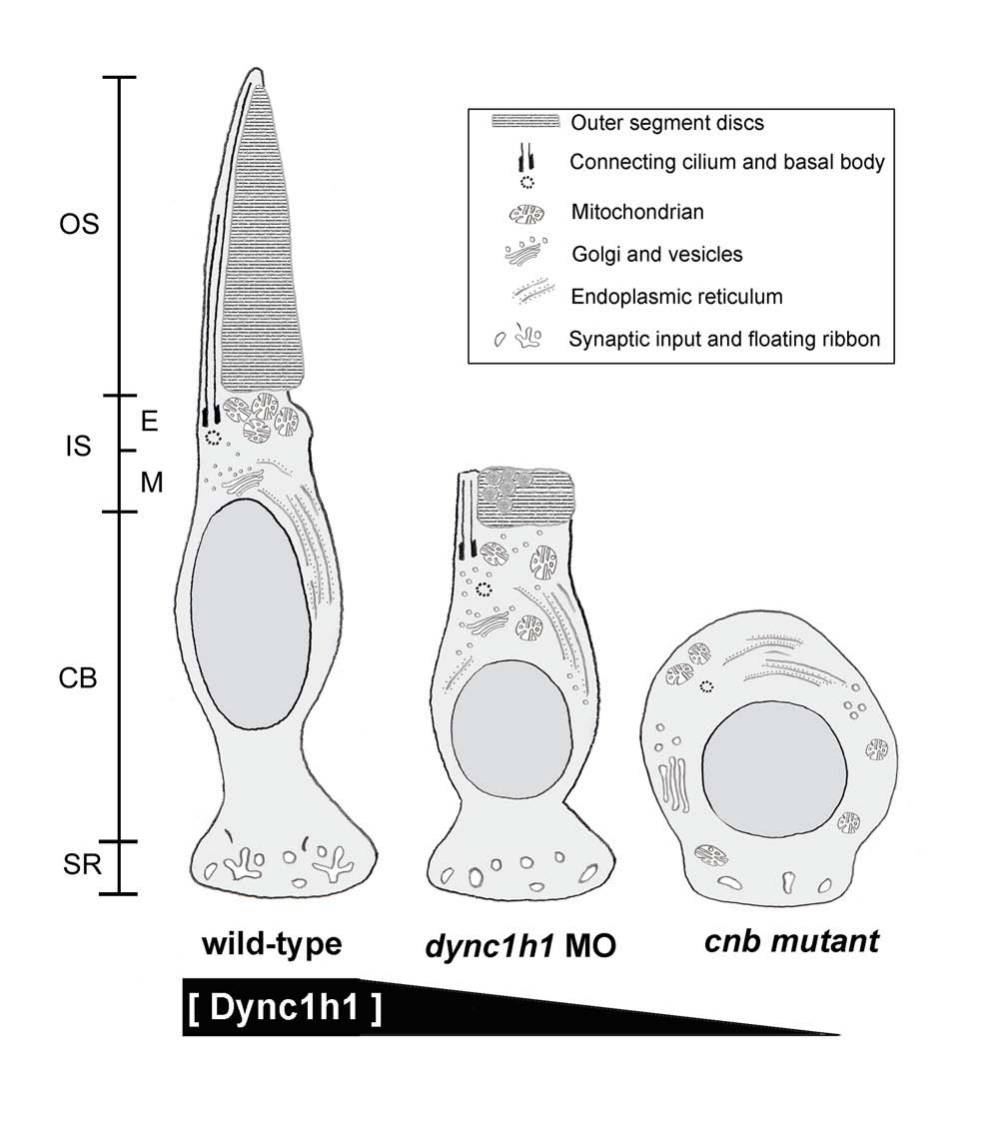
**Schematic of dose-dependent defects observed with loss of Dync1h1**. Illustrations of 3-dpf zebrfish cone photoreceptors from wild-type, low-dose *dync1h1 *morphants, and *cnb *mutants. Wild-type photoreceptors typically showed non-vesiculated outer segments, well organized connecting cilium and basal body, tightly packed mitochondria in the ellipsoid region, Golgi apparatus just apical to the nucleus in the myoid region, and numerous inputs and floating ribbons in the synaptic region. Photoreceptors of *dync1h1 *morphants and *cnb *mutants showed a spectrum of phenotypes with the most normal photoreceptors positioned in the central retina and more severely affected photoreceptors at the periphery. Cellular defects included increased incidence of outer segment vesiculation, varying degrees of organelle dispersion and depolarization, and under-development of the synaptic region. OS, outer segment; IS, inner segment; CB cell body; SR, synaptic region; E, ellipsoid region; M, myoid region.

In contrast to the early embryonic lethality observed in mice lacking *dync1h1 *[9], the presence of residual wild-type maternal protein in zebrafish *cnb *mutants and *dync1h1 *morphants permitted extensive analysis of phenotypes associated with photoreceptor development. Diminished Dynein1 function caused by either genetic mutation or morpholino knock-down resulted in severe photoreceptor defects without dramatic consequences on the development of the organism in general. Figure 14 summarizes the dose-dependent defects of *cnb *and *dync1h1 *morphant photoreceptors that we observed. In the cell body, the nucleus had a rounded appearance and the inner segment rarely developed a normal elongated shape. These two phenotypes were previously described in embryos carrying mutations in *dynactin1 *(*mok*) and *dynactin2 *(*ale oko*), subunits of the Dynein1-interacting protein complex Dynactin [7,33,34]. Additional phenotypes in those dynactin mutants included moderate effects on neurogenesis and aberrant Müller glia cell polarity [34,39]. In our study, we focused on photoreceptors and found that strong loss of Dynein1 function resulted in a total failure of outer segment formation in both rods and cones. In contrast to *mok *and *ale oko *mosaic retinas, outer segment formation in *cnb *cells was not rescued when mutant cells were transplanted in a wild-type environment, indicative of a cell-autonomous function for Dynein1 in outer segment development. However, we cannot exclude non-autonomous influences for more subtle phenotypes associated with loss of *dync1h1*, such as the synaptic differentiation defects.

Since photoreceptors appeared to be dose-sensitive to reduced Dynein1 content, titration of *dync1h1 *morpholino allowed us to inspect photoreceptor cells that had outer segment development. These hypomorphic Dynein1 photoreceptors showed inner segment defects characterized by the abnormal accumulation of vesicles in the ellipsoid region and around the basal body, suggesting post-Golgi trafficking defects. Centrioles were often positioned further from the base of the connecting cilium, which may reflect a generalized disorganization of inner segment polarity. Polarization defects were also indicated by altered localization of multiple organelles, including Golgi and mitochondria. The lack of normal polarization could also be responsible for the failure to form proper synaptic terminals, but could also reflect defects in innervating cell types. Because peripheral *cnb *cells presented the strongest phenotype, a polarization defect could explain the overall rounded shape of these later-born photoreceptors, which are predicted to have the least amount of maternal Dync1h1 protein.

Interestingly, the lowest doses of *dync1h1 *morpholino allowed for outer segment development, but often resulted in significant disorganization of outer segment discs and accumulation of vesicle-like structures. This phenotype also occurred in wild-type cells, but at a lower proportion and to a greater severity (Table 2, Figure 9). We are not sure of the basis for this vesiculation phenotype, but several scenarios are possible. It may be that developing photoreceptors are sensitive to fixation artifacts or simply progress through a disorganized state before assuming a more regular, mature morphology. In this scenario, loss of Dynein1 function may simply delay outer segment development, increasing the proportion of artifacts or developmental disorganization. Alternatively, Dynein1 may be required in the inner segment for trafficking of essential outer segment components. Indeed, enlarged abnormal outer segments were previously reported in mice deficient in Rom-1 and Peripherin [41,42], two proteins required to form and maintain the disc rim structures. Several lines of evidence indicate that an imbalance in the composition of these components in mature discs, and presumably their transport, result in the disruption of disc maintenance. In our prior studies of loss of KIF17, an anterograde IFT motor, we found similar outer segment defects, including accumulation of vesicle-like material in the outer segment [43,44].

A third possible explanation for the outer segment disorganization is that Dynein1 may function directly within the outer segment for maintenance purposes. This possibility is also consistent with outer segment localization of Dynein1. Based on loss of function studies in *Chlamydomonas *and *C. elegans*, as well as subcellular localization studies in mammals, Dynein2 has been proposed as the 'retrograde' IFT motor [17,19,22]. Recent studies of zebrafish embryos lacking Dynein2 function showed cilia defects consistent with a block in retrograde IFT, but in photoreceptor cells the phenotype was relatively normal [18]. One intriguing possibility is that both Dynein1 and Dynein2 function in retrograde transport to maintain photoreceptor outer segments. Finally, it is also possible that a combination of these direct and indirect effects conspire to yield the outer segment phenotypes associated with partial loss of Dynein1 function.

Irrespective of the exact mechanism, our data support an essential (direct or indirect) role for Dynein1 in outer segment development and maintenance. We have shown that Dync1h1 localizes to the ciliary axoneme of outer segments and hypomorphic loss of function conditions showed specific outer segment defects. These data are consistent with both inner and outer segment roles for Dynein1. Intriguingly, the outer segment requirement of Dynein1 appears to be independent of Dynactin function as knock-down of both *dynactin1a*/*1b *subunits resulted in relatively mild phenotypes. Loss of Dynactin 1a/1b resulted solely in mild inner segment polarity and organelle positioning defects. Together, our results support a Dynactin-dependent role for Dynein1 in mitochondria, endoplasmic reticulum/Golgi and centriole positioning within the inner segment and a Dynactin-independent role for Dynein1 in outer segment function. A lack of effect on outer segment development with total loss of Dynactin1 was surprising in light of our finding that Dynactin1, like Dynein1, localized to the ciliary axoneme of the outer segment, in addition to the well-characterized inner segment expression. Potentially, Dynactin1 may normally augment Dynein1 functions in the outer segment, but its presence is not essential for Dynein1 function in this compartment of the photoreceptor.

## Conclusions

In this study we show that the zebrafish *cnb *mutation is due to a nonsense, loss-of-function mutation in the *dync1h1 *gene. Analysis of photoreceptor development implicates cytoplasmic Dynein1 in multiple cellular functions, including organelle positioning, post-Golgi vesicle trafficking, and proper outer segment morphogenesis. Interestingly, titrated knock-down of *dync1h1 *indicated that outer segment morphogenesis was affected in some photoreceptors that showed normal inner segments. These data, combined with localization of Dynein1 components to outer segment axonemes, suggest that Dynein1 may have direct and essential outer segment functions. Our studies provide strong rationale for detailed analyses of microtubule-based motors in photoreceptor development and disease.

## Methods

### Animal husbandry

Zebrafish embryos were raised at 28.5°C and staged according to criteria described by Kimmel and colleagues [45]. Phenylthiourea was applied to embryos to prevent melanization when necessary. All experiments were approved and conducted in accordance with the guidelines set forth by the Institutional Animal Care and Use Committee of the Medical College of Wisconsin.

### Transgenic and mutant lines

Transgenic and mutant lines included:*cannonball*/*dync1h1*^*mw*20 ^(this study); hi3684 (direct data submission to The Zebrafish Model Organism Database [46]); *Tg(Xlrho:*EGFP)^*fl*1 ^[27]; and Tg(1.3*xops*:xRhoCT44-GFP)^a125 ^[40].

### Antibodies

Antibodies included: mouse monoclonal anti-acetylated-α-tubulin (Sigma-Aldrich, St Louis, MO, USA); rabbit polyclonal anti-human Dync1h1 (amino-terminal 321 amino acids; serum #46, gift from Dr Richard Vallee, Columbia University); rabbit polyclonal anti-human Dync1h1 (carboxy-terminal 400 amino acids; 12345-1, ProteinTech, Chicago, IL, USA); rabbit polyclonal anti-rat Dync1h1 (amino acids 4,320 to 4,644; Dynein HC, R-325: sc-9115, Santa Cruz Biotechnology, Santa Cruz, CA, USA); rabbit polyclonal anti-Dync1i1 (gift from Dr Richard Vallee, Columbia University); rabbit polyclonal anti-Dynct1/p150 antibody (gift from Dr Kevin Vaughan, University of Notre Dame); mouse monoclonal anti-SV2 (Developmental Studies Hybridoma Bank).

### Morpholino knockdown

Morpholino oligonucleotides (GeneTools, Inc., Philomath, OR) were targeted to splice site junctions (SP) or the translation start site (ATG) for *dync1h1 *or *dnct1b*: *dync1h1 *ATG MO, 5'-CGCCGCTGTCAGATTTCCTACAC-3'; *dnct1b *ATG MO 5'-TCTGAACTCATTCTGCTGCTGCCGC-3'; dnct1b SP MO, 5'-TCTATAACCATGTTTGACCTTGCTG-3'. Morpholinos were injected into one- to two-cell stage embryos at 10 nl volumes. Specific amounts injected varied from 2 to 10 ng total morpholino per embryo and are described in the text.

### Light microscopy

Embryos were dechorionated and fixed overnight at 4°C in 2.5% gluteraldehyde/1% paraformaldehyde in phosphate buffered sucrose, pH7.4. The next morning embryos were dehydrated and infused with Epon. Transverse sections 1 μm thick were heat-mounted on gelatin coated glass slides, and stained with 1% toluidine blue. Images were captured using a Nikon5700 digital camera mounted on a Nikon E600 compound microscope.

### Transmission electron microscopy

Fish were fixed in primary fixative and washed as for light micorscopy. Specimens were then post-fixed with 1% osmium tetroxide on ice for 1 hour to preserve membranes. Fish were dehydrated through a methanol series and acetonitrile and infiltrated with EMbed-812/Araldyte resin mixture. Ultrathin sections (60 to 70 nm) were collected on coated grids and stained with uranyl acetate and lead citrate for contrast. Images were captured digitally using Hitachi H600 TEM.

### Transmission electron microscopy morphometrics

For quantitative TEM analysis, 5,000× magnification images were collected in the central and peripheral regions of the retina. Central was defined as the 50% middle arc length and the periphery as the 25% arc lengths at each retinal edge from where the marginal zone ended and cellular differentiation was evident. For each condition a minimum of three fields of view from each eye was evaluated and at least three eyes (from three different embryos) were scored. For each field of view the total number of photoreceptor cells was counted and total photoreceptor area as well as outer segment area was measured. All cell areas were measured using the Region Measurement function in MetaMorph software (MDS Analytical Technologies, Toronto, Canada). Finally, each cell was scored for outer segment vesiculation, depolarization (rounded shape, inappropriate organelle positioning), or as being apoptotic (condensed nuclei).

### Western blotting and inner/outer segment extract preparation

The DEPC fraction was prepared from dark adapted bovine retinae as previously described [47].

For zebrafish extracts, embryos were terminally anesthetized and lysed using a plastic dounce fitted to a 1.8 ml microfuge tube. Embryos were homogenized in 200 to 400 μL of lysis buffer (1% Triton X-100/phosphate-buffered saline with 1× Protease Inhibitor Cocktail (Sigma, Cat#P8340)). A small aliquot of the homogenate was taken for protein concentration estimation and the rest added to an equal volume of sample buffer (2× Laemmli buffer). Samples were then boiled for 5 minutes and stored at -20°C until being subjected to PAGE using a 4 to 12% gradient SDS Ready gel (Bio-Rad, Hercules, CA, USA), Following PAGE, proteins were transferred to PVDF membrane using a semi-dry transfer apparatus. Blots were probed through standard methodology.

### Immunolabeling

Adult zebrafish isolated inner segment/outer segment preparations and immunolabelings were performed as described previously using -20°C methonal:acetone (1:1) as the fixative [43]. Specimens for cryosections were fixed with 4°C, 4% paraformaldehyde and processed as previously described [48]. All images were obtained using confocal microscopy.

### Mosaic analysis

Blastulae transplantation was performed as previously described to generate chimeric embryos [48]. Four separate experiments were carried out to achieve n = 10 embryos for both wild-type donor/wild-type host and *cnb *donor/wild-type host.

### Generation of -*3.2 kb gnat2*:Man2a(1-100aa)-GFP construct

Gateway cloning technology (Invitrogen, Carlsbad, CA) was used in tandem with the Tol2 kit [49] to generate a 5 prime entry clone containing a previously characterized cone-specific photoreceptor promoter sequence (-3.2 kb of *gnat2*) [50]. A middle entry clone was then generated by PCR to incorporate the first 100 codons of zebrafish mannosidase2A, which is known to localize specifically to the Golgi apparatus [51]. Finally, a 3 prime entry clone containing GFP was used to add an in-frame carboxy-terminal fusion with the fluorescent protein.

## Abbreviations

*cnb*: *cannonball*; dfp: days post-fertilization; *dctn1a*: *dynactin1a*; DEPC: detergent-extracted photoreceptor cytoskeleton; *dync1h1*: *cytoplasmic dynein heavy chain 1*; EGFP: enhanced green fluorescent protein; IFT: intraflagellar transport; TEM: transmission electron microscopy.

## Competing interests

The authors declare that they have no competing interests.

## Authors' contributions

CI contributed to experiments and wrote the initial manuscript. LMB contributed to experiments and helped edit the manuscript. AA isolated and characterized the hi3684 mutant and helped edit the manuscript. JCB and BAL supervised the study design and experiments, contributed to experiments, and revised the manuscript.

## Supplementary Material

Additional file 1 **Dync1h1 antigen alignments**. Amino acid alignments between zebrafish Dync1h1 sequence and **(A) **human DYNC1H1, amino-terminal 321 amino acids, **(B) **human DYNC1H1, carboxy-terminal 400 amino acids, and (C) rat Dync1h1 (amino acids 4,320 to 4,644). For each, zebrafish sequence is shown at the top, the sequences used to generate the antibodies used in our studies are shown at the bottom, and the conserved amino acids are shown in the middle. Click here for file

Additional file 2 **Morpholino knock-down of *dnct1b***. **(A, B) **Ocular histology of control (A) and *dnct1b *(B) morphants at 3 dpf. **(C) **RT-PCR analysis of *dnct1b *morpholino-injected and control morpholino-injected embryos indicating efficient targeting of the transcript. Primers in the top image bind to regions flanking the morpholino-targeted splice junction. **(D) **RT-PCR analysis of *dnct1b *and control morphants. Primers in the top image bind to the 5' untranslated region of *dnct1b*. Bottom images in (C, D) show results using control EF1alpha primers that indicate the *dnct1b *morpholino destabilizes the targeted mRNA. The age of embryos used to make cDNA is shown at the top of (C, D). Scale bar: 40 μm in (A, B). Click here for file
