# Analysis of a zebrafish *dync1h1 *mutant reveals multiple functions for cytoplasmic dynein 1 during retinal photoreceptor development

**DOI:** 10.1186/1749-8104-5-19

**Published:** 2010-08-06

**Authors:** Christine Insinna, Lisa M Baye, Adam Amsterdam, Joseph C Besharse, Brian A Link

**Affiliations:** 1 Department of Cell Biology, Neurobiology and Anatomy, Medical College of Wisconsin, 8701 Watertown Plank Road, Milwaukee, WI 53226-0509, USA; 2 David H. Koch Institute for Integrative Cancer Research at Massachusetts Institute for Technology, 77 Massachusetts Avenue E17-336, Cambridge, Massachusetts 02138, USA

## Correction

After publication of our recent analysis of the zebrafish *dync1h1 *gene [1], we discovered a typographical error in the Methods. The sequence listed for the *dyn1h1 *ATG morpholino omits 2 nucleotides (in bold below).

The correct sequence is:

*dyn1h1 *ATG morpholino: 5'-CGCCGCTGTCAGA**CA**TTTCCTACAC-3'

The morpholino oligonucleotide that was synthesized and used for our studies was correct and the results were therefore not affected. We apologize for this typographical error.

## References

[B1] Insinna C Baye LM Amsterdam A Besharse JC Link BA Analysis of a zebrafish *dync1h1 *mutant reveals multiple functions for cytoplasmic dynein 1 during retinal photoreceptor development Neural Development 2010 5 12 10.1186/1749-8104-5-12 20412557 PMC2880287

