# Supplementary figures and images for: Analysis of a zebrafish dync1h1 mutant reveals multiple functions for cytoplasmic dynein 1 during retinal photoreceptor development
Source: Neural Dev. 2010 Apr 22;5:12. doi: 10.1186/1749-8104-5-12 (PMC2880287; doi:10.1186/1749-8104-5-12)

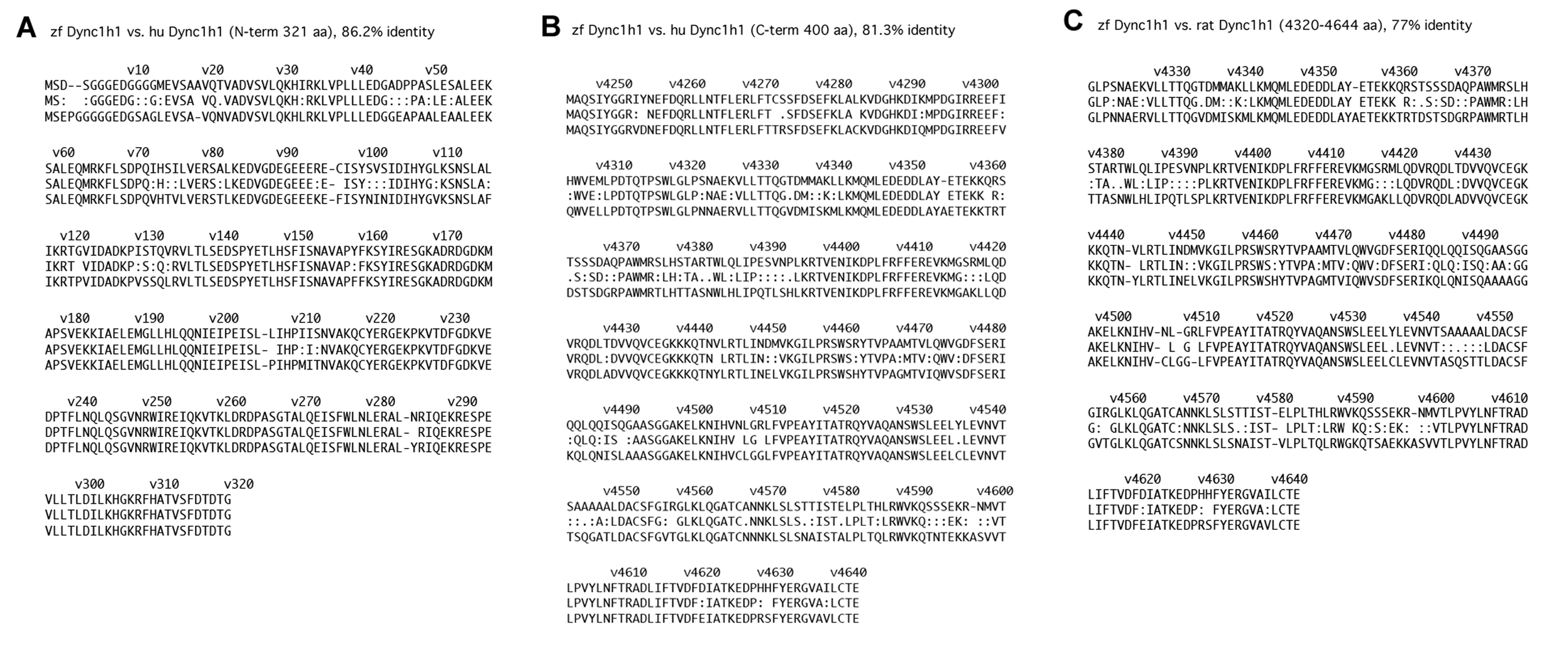

Supplement: Additional file 1 — Dync1h1 antigen alignments. Amino acid alignments between zebrafish Dync1h1 sequence and (A) human DYNC1H1, amino-terminal 321 amino acids, (B) human DYNC1H1, carboxy-terminal 400 amino acids, and (C) rat Dync1h1 (amino acids 4,320 to 4,644). For each, zebrafish sequence is shown at the top, the sequences used to generate the antibodies used in our studies are shown at the bottom, and the conserved amino acids are shown in the middle. [file 1749-8104-5-12-S1.TIFF]

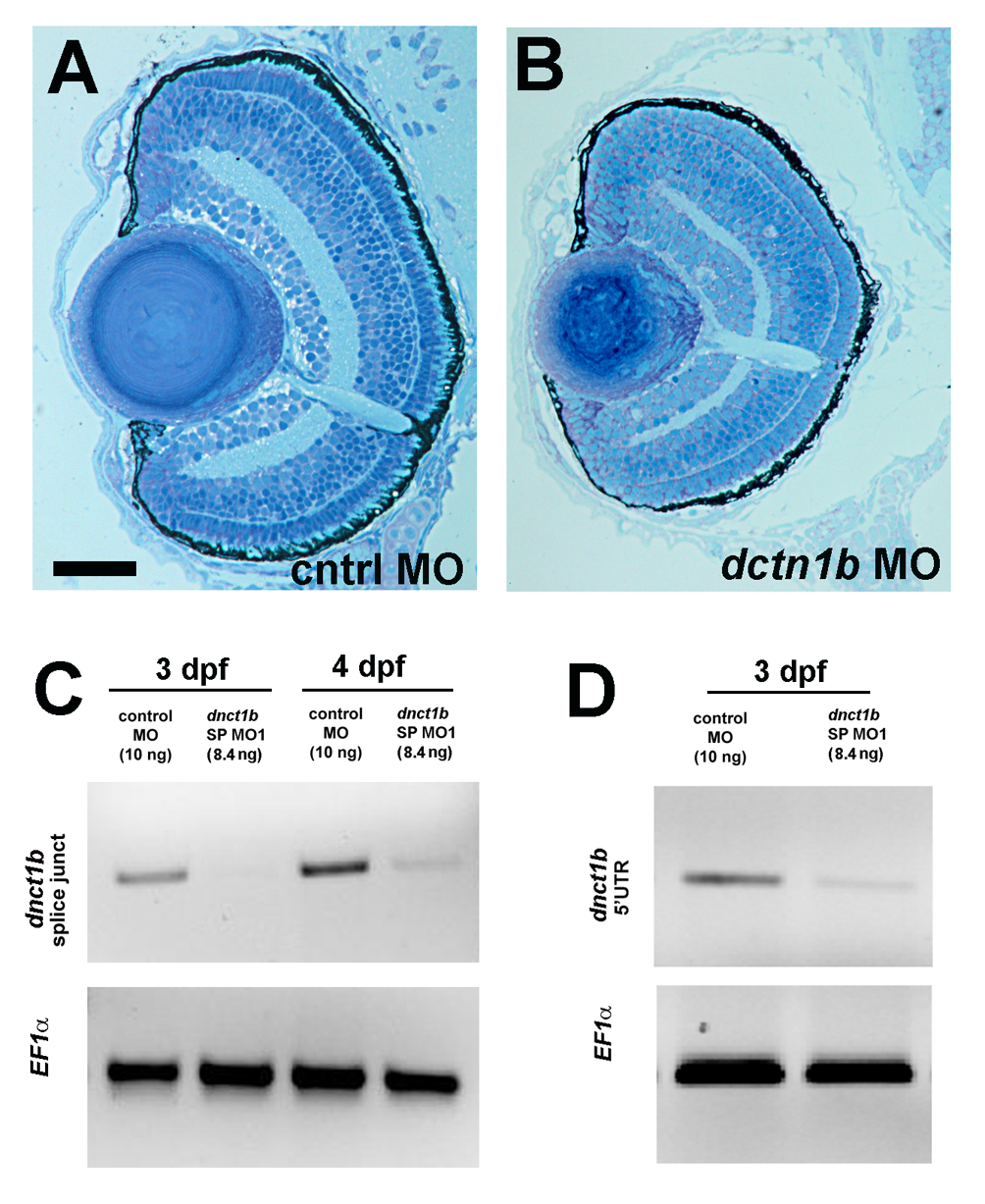

Supplement: Additional file 2 — Morpholino knock-down of dnct1b. (A, B) Ocular histology of control (A) and dnct1b (B) morphants at 3 dpf. (C) RT-PCR analysis of dnct1b morpholino-injected and control morpholino-injected embryos indicating efficient targeting of the transcript. Primers in the top image bind to regions flanking the morpholino-targeted splice junction. (D) RT-PCR analysis of dnct1b and control morphants. Primers in the top image bind to the 5' untranslated region of dnct1b. Bottom images in (C, D) show results using control EF1alpha primers that indicate the dnct1b morpholino destabilizes the targeted mRNA. The age of embryos used to make cDNA is shown at the top of (C, D). Scale bar: 40 μm in (A, B). [file 1749-8104-5-12-S2.TIFF]
